# Clinico-serological associations of urinary activated leukocyte cell adhesion molecule in systemic lupus erythematosus and lupus nephritis

**DOI:** 10.1007/s10067-024-06883-x

**Published:** 2024-01-31

**Authors:** Arwa S. Amer, Samia M. Abdel moneam, Nashwa I. Hashaad, Eman M. Yousef, Dalia M. Abd El-Hassib

**Affiliations:** 1 Rheumatology, Rehabilitation, and Physical Medicine, Faculty of Medicine, Benha University, Fareed Nada Street, Benha, Qalubiya Governorate Arab Republic of Egypt 13511; 2 Clinical and Chemical Pathology, Faculty of Medicine, Benha University, Fareed Nada Street, Benha, Qalubiya Governorate Arab Republic of Egypt 13511

**Keywords:** Lupus nephritis, rSLEDAI, SLE, SLEDAI, urinary ALCAM

## Abstract

**Background:**

Lupus nephritis (LN) is one of the major complications associated with Systemic Lupus Erythematosus (SLE). Activated leukocyte cell adhesion molecule (ALCAM or CD166) is a promising urine biomarker that binds to CD6, a receptor found on lymphocytes. This binding results in T-cell activation, proliferation, and recruitment, which causes tissue inflammation and may explain the pathophysiology of LN.

**Aim of work:**

Investigate the urinary ALCAM level in SLE, study its relationship to disease activity, and clarify the association with LN activity and histopathology.

**Patients and methods:**

A case–control study was performed on 60 patients with SLE and 20 matched controls. The SLE disease activity index (SLEDAI) and the activity of renal disease (rSLEDAI) were evaluated. Renal biopsy and uALCAM levels were also investigated.

**Results:**

Urinary ALCAM levels were higher significantly in active LN patients than inactive LN patients, active and inactive non-LN SLE, and the control group (p < 0.001). The cut-off value for identifying active and inactive LN was above 270 ng/mg (p < 0.001). ALCAM levels were greater in proliferative (class III, IV, and IV/V) than in non-proliferative (class II and V) LN (p < 0.001). ALCAM exhibited high positive correlations with SLEDAI and rSLEDAI (p < 0.001 each) and negative significant correlations with C3 (p < 0.001) and C4 (p = 0.005).

**Conclusion:**

Urinary ALCAM is a sensitive biomarker evaluating LN in SLE patients. Levels above 270 ng/mg can help distinguish between active and inactive LN. ALCAM levels are correlated positively with SLEDAI and rSLEDAI but have a negative correlation with C3 and C4.

**Table:** 

**Key Points** • *Urinary ALCAM shows promise as a biomarker for evaluating kidney dysfunction in SLE patients.* • *It is a non-invasive marker that can differentiate between proliferative and non-proliferative LN.* • *A urinary ALCAM level above 270 ng/mg can indicate active LN, while lower levels indicate inactive LN.* • *Urinary ALCAM levels are correlated positively with SLEDAI and rSLEDAI scores but correlated negatively with C3 and C4.*

**Supplementary Information:**

The online version contains supplementary material available at 10.1007/s10067-024-06883-x.

## Introduction

The hallmarks of SLE include a vast array of autoantibodies that stimulate the immune system to attack the body's tissues, causing damage to several systems and organs [1]. Lupus nephritis is one of the major complications of SLE that primarily affects the kidneys, leading to significant morbidity [2]. Immune complexes containing nucleic acids are deposited in the renal glomeruli, leading to kidney injury through inflammatory, proliferative, and fibrotic pathways [3]. Although kidney biopsy is currently the most reliable way to diagnose and evaluate LN, it is an invasive procedure that carries various risks. Therefore, it is important to get a non-invasive and accessible method to monitor the progression of LN and evaluate the effectiveness of immunosuppressive treatments. This will help determine the treatment outcome and improve the overall management of LN [4].

Activated leukocyte cell adhesion molecule (ALCAM or CD166) is a promising urine biomarker for LN [5]. It is a surface-transmembrane-glycoprotein and one of the superfamily immunoglobulins that is mainly expressed on dendritic cells, but it is also found on cells that present antigens like macrophages and B cells. ALCAM functions as a CD6 ligand [6]. CD6 is a receptor found on the surface of lymphocytes, which plays a crucial role in the development and activation of T cells. When the CD6 receptor binds to its ligand ALCAM, it acts as a co-stimulatory molecule that enhances the activation, proliferation, and recruitment of T-cells. These events lead to tissue inflammation, and this may be the pathophysiology of LN [7]. Anti-CD6 monoclonal antibodies block ALCAM interaction, inhibiting T cell activation and trafficking, and reducing concentrations of T lymphocytes that infiltrate the kidneys, leading to a reduction in the renal impairment degree and LN [8]. The purpose of the research was to examine the urinary level of ALCAM in SLE, investigate its relationship to SLE disease activity, and clarify its association with LN activity and histopathology.

## Patients and methods

### Study design

A case–control study was performed on sixty individuals with SLE. They were split into two subgroups depending on whether they had LN or not. The first subgroup (Ia) included SLE patients without LN, while the second subgroup (Ib) included SLE patients with LN. Twenty controls were matched by age and gender (group II).

### Methods

The research was carried out between September 2021 and September 2022, with patients gathered from the inpatient department of rheumatology, rehabilitation, and physical medicine at Benha University Hospitals in Egypt, as well as the outpatient clinic. All patients met the 2019 EULAR/ACR updated SLE categorization criteria [9]. Individuals who were less than 18 years of age or had autoimmune diseases other than SLE were not included in the study. The Benha University Ethics Committee, Egypt, has accepted this study; the approval number is **MD.7.7.2021**. Every patient provided informed consent before participating.

According to the total clinical SLEDAI score and the rSLEDAI index, patients were categorized into four groups; 15 patients with active LN (rSLEDAI score ≥ 4), 15 active non-renal SLE patients (SLEDAI score ≥ 6, but rSLEDAI = 0), 15 inactive LN and LN history (rSLEDAI = 0) and 15 patients with an inactive non-renal SLE (SLEDAI score = 0).

### Clinical assessment

A thorough medical history was gathered, and a clinical examination was conducted. SLE disease activity was evaluated using SLEDAI-2 K, while renal activity was assessed using rSLEDAI [10]. SLEDAI scores were categorized as low (1–5), moderate (6–10), high (11–19), and extremely high (≥ 20). Renal SLEDAI is made up of the SLEDAI-2 K's four elements related to the kidneys (proteinuria, pyuria, hematuria, and urine casts), and each one is worth 4 points; hence, the score can vary from 0 (not-active renal illness) to 16.

### Laboratory investigations

Complete blood count (CBC), erythrocyte sedimentation rate (ESR), C-reactive protein, complement proteins 3 and 4 (C3 and C4), antinuclear antibodies (ANAs), anti-double-stranded DNA antibodies (anti-dsDNAs), renal function tests, 24-h urinary protein, and uALCAM level. Levels of uALCAM were investigated utilizing a commercial human enzyme-linked immunosorbent assay (ELISA) kit (Bioassay technology laboratories, Shanghai Korain Biotech, Cat.No E0202Hu) according to the manufacturer's instructions.

#### Renal biopsy

A kidney biopsy is carried out by a radiology consultant at the radiology department when there is proteinuria more than or equal to 500 mg/24-h, persistent hematuria or pyuria, and when all other potential causes have been ruled out. It is also done when there is unexplained renal insufficiency and a normal urinalysis [11]. A biopsy was taken under CT supervision using a true-cut needle biopsy. According to the International Society of Nephrology/Renal Pathology Society (ISN/RPS), the predominant histopathological feature is as follows: class I is minimum mesangial lupus nephritis; class II is mesangial proliferative lupus nephritis; class III is focal lupus nephritis; class IV is diffuse segmental (IV-S) or global (IV-G) lupus nephritis; class V is membranous lupus nephritis and class VI is advanced sclerosing lupus nephritis. [12]**.**

### Statistical analysis

Data were shown as mean, standard deviation (SD), and standard error (SE), with maximum and minimum values reported. The student's t-test was utilized to determine whether the variance in the means of the two study groups was statistically significant, Mann–Whitney test was utilized to evaluate the statistical significance of variation in a non-parametric variance between two research groups. The statistical significance of the variation in a non-parametric variable between more than 2 groups was evaluated using the Kruskal–Wallis test. The receiver operating characteristic (ROC) Curve was utilized to represent the overall performance of a diagnostic test by linking the coordinate points with “1 – specificity” (= false positive rate) as the x-axis and “sensitivity” as the y-axis. The area under the ROC curve (AUC) was used to measure the overall performance of a diagnostic test. The test's overall performance improves as the AUC value increases. The optimal cut-off value was found by maximizing sensitivity and specificity, Logistic regression analysis was utilized to predict risk variables. P-values were regarded as significant if it is < 0.05. Version 22 of the SPSS. (IBM Corp., Armonk, New York, USA) program was utilized for all analyses.

**The sample size** was determined utilizing Stata Corp. 2021. Stata Statistical Software: Release 17. College Station, TX: Stata Corp LLC. Using the t-test model, and ROC curve model; the required minimal sample size is 40 subjects (20 SLE cases and 20 healthy control subjects), using alpha error (the probability of a type I error) of 5% and power (the probability of not making a type II error) of 80%. The SLE group was increased to 60 patients to boost the study's power.

## Results

This research involved 60 patients with SLE, out of which 47 were females (78.3%) and 13 were males (21.7%). The patients' average age was: 29.27 ± 5.07 years. A healthy control group was also incorporated into the research, with similar age and gender. Patients with SLE, both with and without Lupus Nephritis (LN), had an average illness duration of 5.83 ± 0.61 and 5.63 ± 0.61 years respectively.

**Fig. 1:**
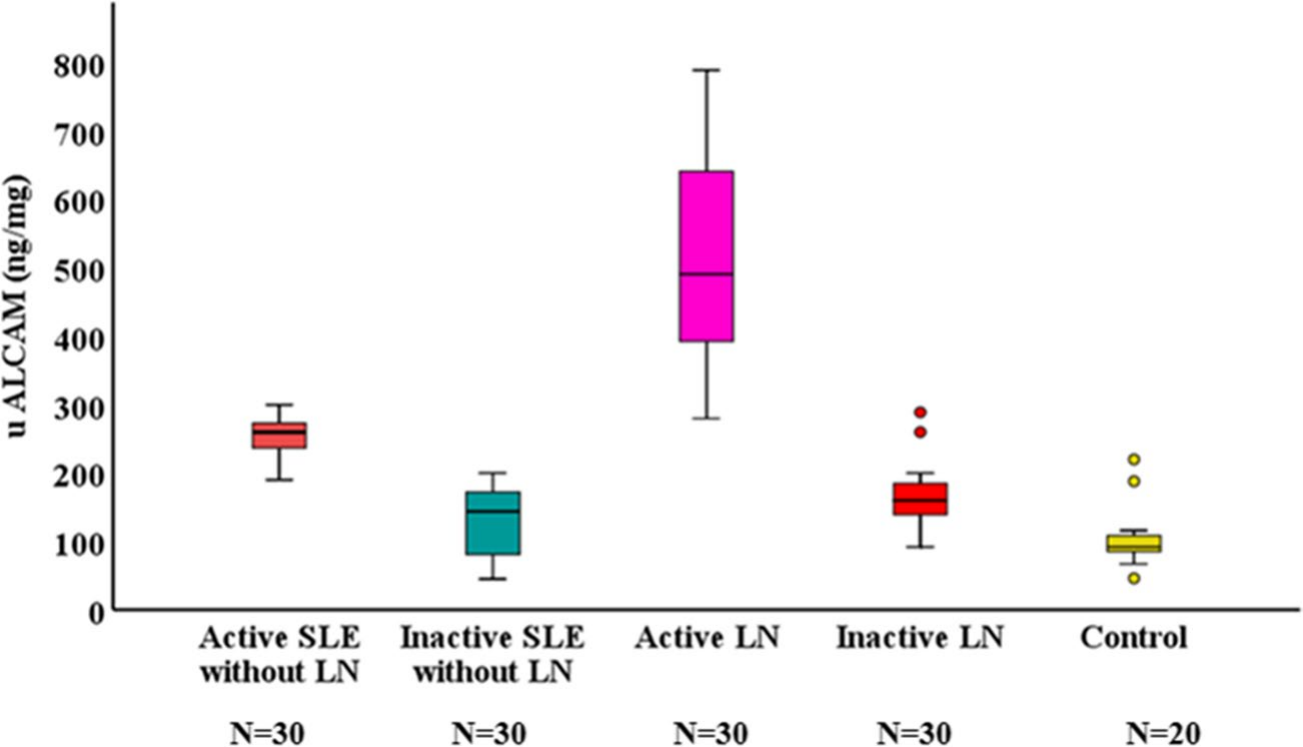
Boxplot for comparison of uALCAM levels among different subgroups

**Fig. 2:**
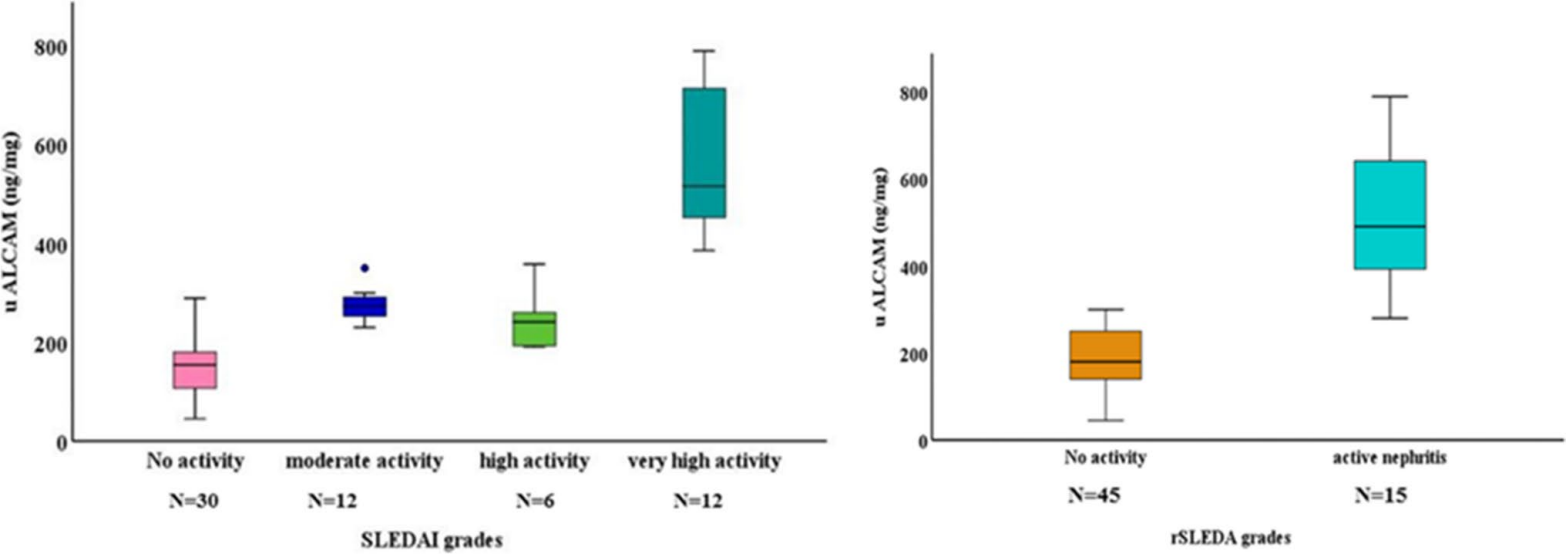
Boxplot for comparison of uALCAM levels among SLEDAI and rSLEDAI grades

Active lupus nephritis patients have significantly higher uALCAM levels than non-renal active SLE patients, inactive LN, non-renal inactive SLE patients, or controls (p < 0.001 each); Fig. 1. Furthermore, patients with high disease activity and active LN had higher uALCAM levels and the levels differed significantly among SLEDAI and rSLEDAI grades (p < 0.001 each), as shown in Fig. 2. The uALCAM level is also observed to vary significantly among renal biopsy classes (p < 0.001). It was highest in classes III, IV, and IV/V (proliferative LN); whereas, it was lowest in classes II and V (non-proliferative LN) as detailed in Table 1.

**Table 1:** Comparison between uALCAM levels among renal biopsy classes

	N	u ALCAM					Test (p)
		Mean ± SE		Median	Range		
Biopsy not indicated	45	182.9 ± 10.41		180.0	45.0 – 300.0		Mann-U = 670.5 p < 0.001*
Biopsy indicated	15	515.8 ± 41.09		491.0	280.0 – 790.0		
Renal biopsy classes							H = 33 P < 0.001*
II	1	280.0		280	280	280	
III	4	532.0	90.1	476	386	790	
IV	6	580.7	44.9	548	470	725	
V	2	354.0	4.0	354	350	358	
IV/V	2	568.5	168.5	569	400	737	

SE. Standard Error, Range: Min. – Max., *: Significant ≤ 0.05

The level of uALCAM was found to vary significantly among different clinical features of SLE. It was observed to be considerably higher in cases of malar rash, arthritis, nephritis, anemia, leucopenia, and anti-dsDNA (p < 0.001 each). Similarly, it was found to be statistically related to fever, alopecia, pericarditis (p = 0.001 each), pleurisy (p = 0.002), lymphadenopathy (p = 0.01), oral ulcer (p = 0.012), thrombocytopenia (p = 0.039), and myositis (p = 0.047).

**Fig. 3:**
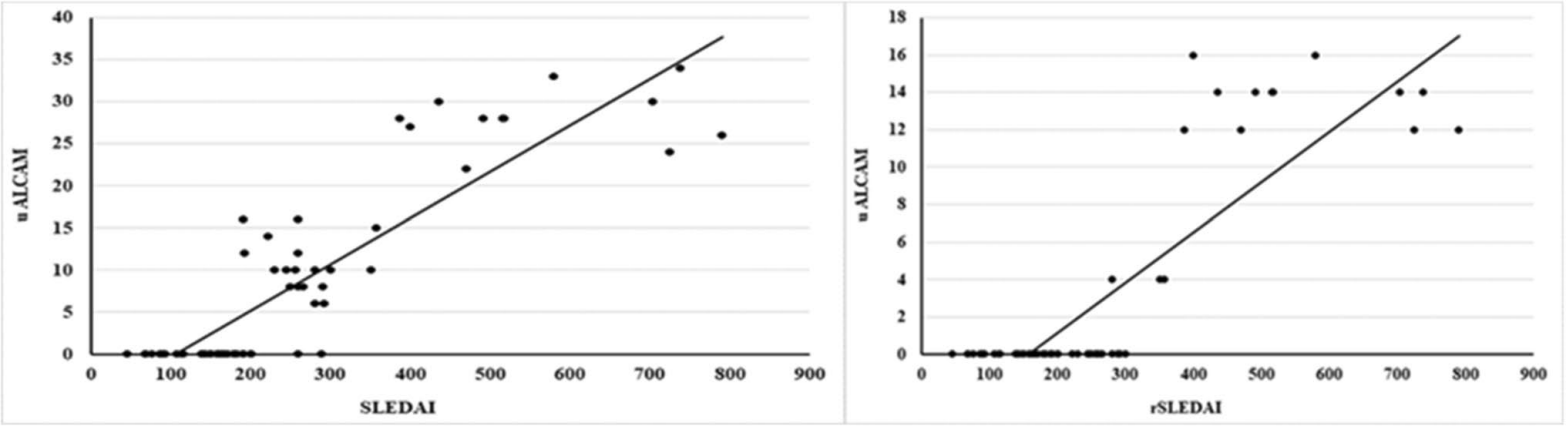
Correlation of uALCAM levels with SLEDAI and rSLEDAI among SLE patients

The levels of uALCAM were strongly positively correlated with disease duration, ESR, 24-h urinary protein, hematuria, SLEDAI, and rSLEDAI scores (p < 0.001 each) Fig. 3, pyuria, and urea level (p = 0.001 each), as well as serum creatinine (p = 0.028). However, there were strongly negative correlations of ALCAM levels with serum albumin and C3 (p < 0.001 each) and a statistically negative correlation with C4 (p = 0.005).

**Fig. 4:**
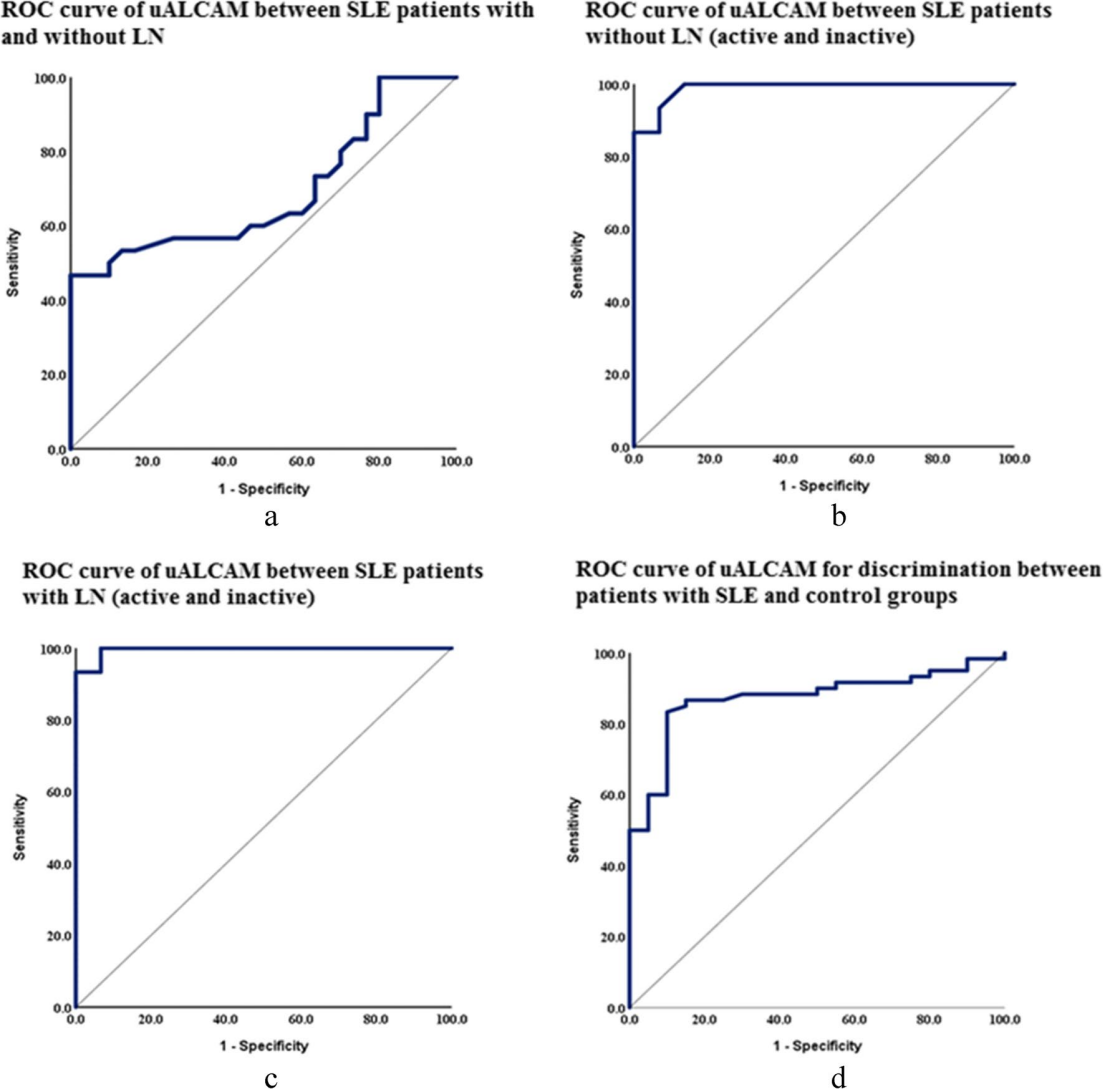
ROC of uALCAM levels between the studied groups and subgroups. **a:** ROC curve of uALCAM between SLE patients with and without LN; **b:** ROC curve of uALCAM between SLE patients without LN (active and inactive); **c:** ROC curve of uALCAM between SLE patients with LN (active and inactive); **d:** ROC curve of uALCAM between SLE and control groups

Based on the ROC curve, the uALCAM level cutoff for distinguishing between SLE patients without and with LN is more than 196.5 ng/mg. This resulted in a sensitivity of 60% and specificity of 53.3%, and a statistically significant variation (p = 0.012), shown in Fig. 4a. Similarly, the cutoff value for differentiating between active and inactive non-renal SLE patients is greater than 186 ng/mg, with a sensitivity of 100% and specificity of 86.6% and a highly significant difference (p < 0.001), as shown in Fig. 4b. With a sensitivity of 100% and specificity of 93.3%, the uALCAM level cutoff was more than 270 ng/mg to distinguish between active and inactive LN. The difference was highly significant (AUC = 0.996, 95% CI = 0.981–1.0, p < 0.001), shown in Fig. 4c. Finally, the cutoff value for differentiating between SLE and control was greater than 112.5 ng/mg, with a sensitivity of 86.7% and specificity of 85% with a strongly significant variance (p < 0.001), as shown in Fig. 4d.

Regression analyses indicated that a higher 24-h urinary protein, SLEDAI, rSLEDAI, and uALCAM levels were unfavorable risk predictors for the occurrence and activity of LN (supplementary Table 1 & 2).

## Discussion

Although there have been substantial advancements in understanding the causes of LN and treatment options, remission is only achieved in 50–70% of patients. Therefore, it is essential to screen all patients with SLE for LN [13]. In Lupus Nephritis, the ALCAM, also known as CD166, plays a pathogenic role. This has been demonstrated by blocking the ALCAM-CD6 pathway with anti-CD6 monoclonal antibodies, resulting in a reduction in T cell count infiltrating the kidneys, which leads to a decrease in renal impairment and LN severity [14].

In this study, levels of uALCAM were higher in active LN patients than inactive LN patients, active and inactive non-LN SLE, and controls. Studies carried out by Der et al. [15], Ding et al. [16], Parodis et al. [17], Lei et al.[18], and Chalmer et al. [19] supported this finding and all support the hypothesis that uALCAM is a crucial renal biomarker, given its expression by the cells and tissues involved in LN [20]. The current study observed higher levels of uALCAM in classes III, IV, and IV/V (proliferative LN) than in classes II and V (non-proliferative LN) and these findings are consistent with previous studies by Chu et al.[21], who also reported elevated levels of uALCAM in subjects with proliferative LN, and Ding et al. [16], who found that uALCAM levels showed a significant increase in classes III and IV (proliferative) than class V (membranous) LN.

This work established the highly statistically significant positive correlation of uALCAM with SLEDAI and rSLEDAI scores which is consistent with the results of Parodis et al.[22]**, **Lei et al. [18]**, **Chalmer et al.[19]**, **Kim et al.[23]**.**

The present research revealed that uALCAM is highly positively correlated with ESR, proteinuria, and anti-dsDNA and positively correlated with serum creatinine. This finding is consistent with the results of previous studies conducted by Kim et al. [23], Stanley et al.[24], and Ding et al. [16]. Kim and colleagues similarly reported a positive correlation between ALCAM and anti-dsDNA, while Stanley et al. found that ALCAM positively correlated with ESR, proteinuria, and anti-dsDNA. Ding et al. also reported a positive correlation between uALCAM and 24-h urinary protein. However, unlike our results, they showed no significant association between uALCAM and anti-dsDNA antibodies or serum creatinine levels. In the present research, negative significant associations were found between uALCAM and serum albumin, hemoglobin percentage, and C3 and C4 levels. These results are similar to those found by Stanley et al. [24], who discovered that uALCAM was inversely associated with levels of C3 and C4 in Chinese and Asian cohorts. However, they found that within the African-American cohort, uALCAM exhibited a poor correlation with C3/C4 levels. Ding et al. [16] also reported a negative correlation between uALCAM and serum albumin, hemoglobin percentage, and C3 level, while they found an insignificant correlation between uALCAM level and serum C4 level. Chu et al. [21] reported that uALCAM significantly correlated negatively with serum C3 and C4 levels.

The present study has shown the specificity of uALCAM level to differentiate between active, and inactive LN is 93.3%. These results are in line with the outcomes of Stanley et al. [24], who discovered that uALCAM had a specificity of 92% when distinguishing between active and inactive LN in their African-American cohort. Similarly, Ding et al. [16] found that the specificity of uALCAM level in active versus inactive LN was 95%.

Using regression analyses, only a higher 24-h urinary protein, SLEDAI, rSLEDAI, and uALCAM levels were considered unfavorable risk predictors for LN occurrence and LN activity.

## Limitations of the study

It is recommended to conduct larger-scale longitudinal research to investigate the role of urinary ALCAM in SLE pathogenesis and its effect on treatment outcomes. The data used in this study was obtained from only one hospital, which may lead to bias in patient selection. All cases in the study were receiving steroids and immunosuppressants, which could affect urinary ALCAM levels. Further studies can focus on anti-CD6 monoclonal antibodies, which block ALCAM interaction and might serve as a novel target therapy for SLE patients and LN.

## Conclusions

Urinary ALCAM is a promising biomarker for distinguishing between proliferative and non-proliferative LN. levels greater than 270 ng/mg can indicate active LN. ALCAM levels correlate positively with SLEDAI and rSLEDAI scores but negatively with C3 and C4 levels. As a pathognomonic factor, uALCAM can be a potential treatment target for SLE and LN.

### Supplementary Information

Below is the link to the electronic supplementary material.

Supplementary file1 (DOCX 21 KB)

## Data Availability

The datasets used and analyzed during the current study are available from the corresponding author on reasonable request.
