# Supplementary material for: Clinico-serological associations of urinary activated leukocyte cell adhesion molecule in systemic lupus erythematosus and lupus nephritis
Source: Clin Rheumatol. 2024 Jan 31;43(3):1015–21. doi: 10.1007/s10067-024-06883-x (PMC10876720; doi:10.1007/s10067-024-06883-x)
Supplement: Supplementary file 1 — Supplementary file1 (DOCX 21 KB) [file 10067_2024_6883_MOESM1_ESM.docx]

**Supplementary table (1): Logistic Regression Analysis for the prediction of LN occurrence**

|  | **Univariate** | | | **Multivariate** | | |
| --- | --- | --- | --- | --- | --- | --- |
|  | **P** | **OR** | **95% C. I** | **P** | **OR** | **95% C. I** |
| Age | 0.442 | 0.975 | 0.915-1.039 |  |  |  |
| Gender | 0.754 | 0.884 | 0.409-1.911 |  |  |  |
| ESR | 0.323 | 1.009 | 0.991 - 1.028 |  |  |  |
| CRP | 0.563 | 0.914 | 0.674 - 1.240 |  |  |  |
| Serum creatinine | 0.024* | 5.495 | 1.254 - 24.086 | 0.412 | 0.686 | 0.279-1.688 |
| Blood Urea | 0.006* | 1.095 | 1.026 - 1.169 | 0.903 | 0.996 | 0.935-1.061 |
| Disease duration | 0.813 | 1.019 | 0.873 - 1.190 |  |  |  |
| 24hr urine Protein | 0.005* | 2.942 | 1.139 - 6.667 | 0.031* | 1.961 | 1.670-2.379 |
| Serum albumin | 0.002* | 0.157 | 0.049 - 0.504 | 0.588 | 0.756 | 0.274-2.081 |
| Anti-dsDNA | 0.006* | 2.765 | 1.343-5.692 | 0.944 | 0.908 | 0.062-3.366 |
| C3 | 0.112 | 0.985 | 0.967 – 1.003 |  |  |  |
| C4 | 0.393 | 0.976 | 0.923 – 1.032 |  |  |  |
| SLEDAI | 0.012* | 1.045 | 1.010-1.081 | 0.016* | 0.826 | 0.707-0.965 |
| rSLEDAI | 0.005* | 1.079 | 1.023-1.138 | 0.028* | 1.462 | 1.041-2.053 |
| uALCAM level | 0.004* | 1.007 | 1.002 – 1.012 | 0.013* | 1.024 | 1.011-1.038 |

OR: odds ratio; CI: confidence interval; Significant: ≤0.05*

**Supplementary table (2):** **Logistic Regression Analysis for prediction of active LN**

|  | Univariate | | | Multivariate | | |
| --- | --- | --- | --- | --- | --- | --- |
|  | **P** | **OR** | **95% C. I** | **P** | **OR** | **95% C. I** |
| Age | 0.140 | 0.898 | 0.810-1.195 |  |  |  |
| Gender | 0.666 | 0.791 | 0.273-2.295 |  |  |  |
| ESR | 0.001* | 1.031 | 1.013-1.051 | 0.070 | 1.037 | 0.997-1.079 |
| CRP | 1.000 | 1.000 | 0.666 – 1.502 |  |  |  |
| Serum creatinine | 0.016* | 1.479 | 1.151 – 5.145 | 0.683 | 0.753 | 0.193-2.938 |
| Urea | 0.005* | 1.136 | 1.040 – 1.241 | 0.596 | 1.029 | 0.925-1.145 |
| Disease duration | 0.005* | 0.490 | 0.298 – 0.806 | 0.287 | 0.860 | 0.651-1.135 |
| 24hr urine Protein | 0.009* | 1.245 | 1.057-1.468 | 0.684 | 0.899 | 0.539-1.500 |
| Serum albumin | 0.003* | 0.047 | 0.006 – 0.350 | 0.500 | 0.532 | 0.085-3.328 |
| C3 | 0.114 | 0.746 | 0.518 – 1.073 |  |  |  |
| C4 | 0.075 | 0.925 | 0.850 – 1.008 |  |  |  |
| Anti-dsDNA | 0.005* | 2.500 | 1.918-3.666 | 0.295 | 2.437 | 0.273-4.091 |
| SLEDAI | 0.002* | 1.082 | 1.029-1.138 | 0.021* | 1.076 | 1.016-1.236 |
| rSLEDAI | 0.003* | 1.164 | 1.054-1.286 | 0.004* | 1.789 | 1.435-2.433 |
| uALCAM level | 0.003* | 1.004 | 1.001-1.006 | 0.028* | 1.523 | 1.194-1.905 |

OR: odds ratio; CI: confidence interval; Significant: ≤0.05*

Regression analyses indicated that a higher 24-hour urinary protein, SLEDAI, rSLEDAI, and uALCAM levels were unfavorable risk predictors for the occurrence and activity of LN (**supplementary tables 1&2)**.
